# Rule-Based Cell Systems Model of Aging using Feedback Loop Motifs Mediated by Stress Responses

**DOI:** 10.1371/journal.pcbi.1000820

**Published:** 2010-06-17

**Authors:** Andres Kriete, William J. Bosl, Glenn Booker

**Affiliations:** 1 School of Biomedical Engineering, Science and Health Systems, Drexel University, Bossone Research Center, Philadelphia, Pennsylvania, United States of America; 2 Harvard Medical School, Children's Hospital Informatics Program, Boston, Massachusetts, United States of America; 3 College of Information Science and Technology, Drexel University, Philadelphia, Pennsylvania, United States of America; University of Washington, United States of America

## Abstract

Investigating the complex systems dynamics of the aging process requires integration of a broad range of cellular processes describing damage and functional decline co-existing with adaptive and protective regulatory mechanisms. We evolve an integrated generic cell network to represent the connectivity of key cellular mechanisms structured into positive and negative feedback loop motifs centrally important for aging. The conceptual network is casted into a fuzzy-logic, hybrid-intelligent framework based on interaction rules assembled from a priori knowledge. Based upon a classical homeostatic representation of cellular energy metabolism, we first demonstrate how positive-feedback loops accelerate damage and decline consistent with a vicious cycle. This model is iteratively extended towards an adaptive response model by incorporating protective negative-feedback loop circuits. Time-lapse simulations of the adaptive response model uncover how transcriptional and translational changes, mediated by stress sensors NF-κB and mTOR, counteract accumulating damage and dysfunction by modulating mitochondrial respiration, metabolic fluxes, biosynthesis, and autophagy, crucial for cellular survival. The model allows consideration of lifespan optimization scenarios with respect to fitness criteria using a sensitivity analysis. Our work establishes a novel extendable and scalable computational approach capable to connect tractable molecular mechanisms with cellular network dynamics underlying the emerging aging phenotype.

## Author Summary

The global process of aging disturbs a broad range of cellular mechanisms in a complex fashion and is not well understood. One important goal of computational approaches in aging is to develop integrated models in terms of a unifying aging theory, predicting progression of aging phenotypes grounded on molecular mechanisms. However, current experimental data incoherently reflects many isolated processes from a large diversity of approaches, biological model systems, and species, which makes such integration a challenging task. In an attempt to close this gap, we iteratively develop a fuzzy-logic cell systems model considering the interplay of damage, metabolism, and signaling by positive and negative feedback-loop motifs using relationships drawn from literature data. Because cellular biodynamics may be considered a complex control system, this approach seems particularly suitable. Here, we demonstrate that rule-based fuzzy-logic models provide semi-quantitative predictions that enhance our understanding of complex and interlocked molecular mechanisms and their implications on the aging physiome.

## Introduction

Approaches to model aging computationally are challenged by an inherent complexity through involvement of a wide range of molecular processes, pathways, oxidative damage, dysfunction of organelles and dysregulation. Therefore, systems level representations combining experimental observations, concepts and computational frameworks have been recognized as a potential avenue to advance our understanding of the aging process [1]–[4]. Biosimulation in general, and computational systems biology more specifically, often starts with a graphical map summarizing conceptual ideas about the underlying interconnectivity of components in biological networks that lead to systems level simulations [5]–[7]. Pathway cartoons depicting the connectivity of proteins in signaling networks are one example and have contributed to the success of computational methods, since these graphs devise computationally tractable procedures relying on reaction-based protein kinetics. In contrast, cell level representations of aging require inclusion of a wider range of descriptors and mechanisms, such as organelle dysfunction, concentration of second messengers, rates of damage, stress sensor signaling, as well as transcriptional and translational alterations. Furthermore, since experimental data in the field of aging is often fragmentary and obtained from different experimental setups and biological model systems, the ability to identify network topologies and underlying mechanistic principles using reverse engineering procedures [8], [9] is currently very limited.

This poses a situation where intuitive, hand-curated models using fuzzy-logic (FL) can provide great help, since they can handle imprecise data of different mechanisms as long as the underlying relationships and rules among all components can be defined [10], [11]. Therefore, fuzzy logic provides a way to arrive at conclusions based upon descriptive, imprecise or noisy input information [11]–[13]. A FL model requires some numerical parameters in order to operate, such as initial values and rate coefficients, but exact values of these numbers are usually not required to explore the dynamics of the model system. Fuzzy logic computing relies on terms of fuzzy quantities, such as meostatic, and with the prescribed logic of interactions complex biological systems of networked reactions or processes can be modeled [14], [15]. However, the FL model is not imprecise; rather, it is a way of modeling and drawing conclusions about system dynamics with imprecise knowledge of the system.

Therefore, our approach is to integrate key mechanisms based on current experimental insight into a broader conceptual graph model, define the underlying connectivity or rules among all components involved, and subsequently perform time-lapse simulations using fuzzy-logic computation. Despite the complexity and variation of aging phenotypes across tissues and species, we hypothesize that the underlying molecular processes of most cell types shares similarities and are conserved, particularly with respect to somatic cells in tissues with low mitotic index representing a major portion of our body mass. Thus, it should be possible to gather and integrate relevant information into a generic model based on the logic of interactions.

Here we assess the utility of feedback loop motifs as logical elements in the conceptual stage of the modeling phase to represent tradeoffs and protective mechanism during aging. Feedback systems have become an important concept for understanding complex behavior of biological and technical systems and can be traced back to Norbert Wiener's seminal work on cybernetic control theory [16]. This concept has been widely recognized to appropriately describe many cellular and physiological processes involving regulation, amplification and inhibition. Feedbacks can be classified into motifs, depending on their function, and may consist of either amplifying positive or inhibiting negative feedback loops. Depending on the amount of feedback, negative-feedback systems stabilize signaling levels, limit transient and maximal output or support adaptation [17], [18]. In complex pathways, such as those underlying the biology of aging and energy metabolism, processes are fine-tuned by mixing different feedback motifs and by the amount of amplification of each component participating in the feedback mechanisms [19], [20].

**Figure 1:**
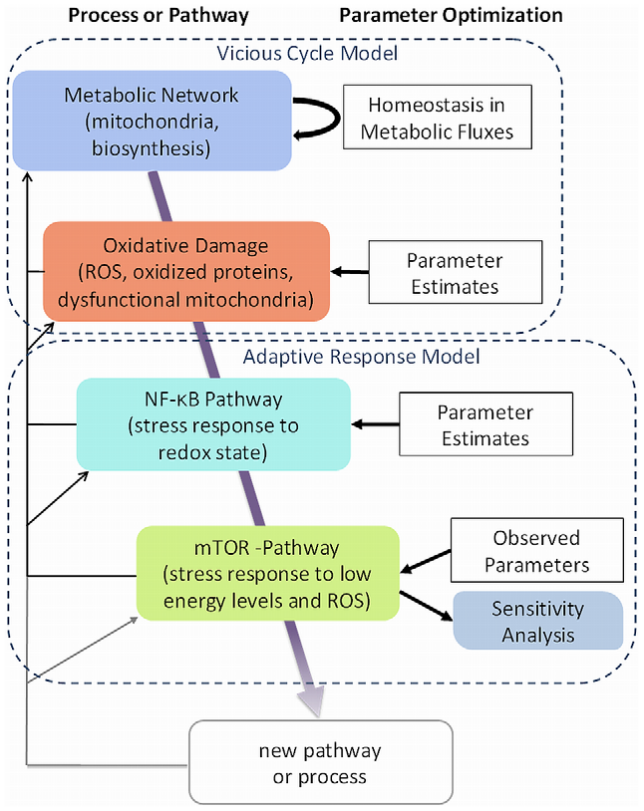
Iterative model development. Overview of the steps taken to assemble a generic cell aging model. The model is instantiated with a combined energy producing (mitochondria) and consuming (biosynthesis) complex and a parameter setting that provides homeostasis in metabolic fluxes, which is disturbed by reactive oxygen species (ROS) as a byproduct of mitochondrial respiration, damaging proteins and organelle function. In the next iterations stress response pathways are assembled into the network topology providing adaptive and regulatory systems feedback. This includes NF-κB, a sensor of oxidative stress, and mTOR, an energy sensor. The resulting model is investigated by a sensitivity analysis. The model can be extended and scaled.

We evolve our model with increasing degrees of complexity and transfer many essential parameters from one stage to the next in order to make model predictions comparable. An overview of the iterative development process is provided in Figure 1. Here we start with a homeostatic model of energy metabolism including mitochondria and biosynthesis. This initial representation is extended to encompass free radical levels, accumulating damage and organelle dysfunction. The model demonstrates accelerating dysfunction throughout the biological system resembling the characteristic of an amplifying positive feedback loop, or vicious cycle (VC), and does not assume strong forces in defense of dysfunction nor activation of adaptive mechanisms to prevent such deleterious processes. However, aging also has recognizable regulatory and adaptive components. For instance, many studies have found genes consistently up- or down-regulated with age, which points to the involvement of crucial transcription factors in cellular alterations. Activated by sensors of intracellular stress, redox-regulated pathways and transcription factors are influential in shaping the transcriptional profile of batteries of genes and potentially play a more crucial role in the change of the behavior of cells than random damage to proteins [21]–[23]. Here we consider two well characterized representatives of such stress pathways, the pro-survival transcription factor Nuclear Factor kappa B (NF-κB), and the energy sensor mammalian target of rapamycin (mTOR), involved in both transcriptional and translational regulation, as provided from referenced studies of human aging. NF-κB is a marker of elevated stress and redox state and known to be constitutively activated by cell-intrinsic “atypical” mechanisms in aging [24], [25]. The kinase mTOR is negatively regulated by energy availability (ATP), with a role in mitochondrial respiration, protein synthesis and autophagy [26]–[28]. In consideration of the relevance of these two stress sensors in aging, we assembled an Adaptive Response (AR) model. The extended network topology is characterized by negative feedback loops mediated by NF-κB and mTOR, which activate defensive mechanisms and mediate metabolic readjustments counteracting an accelerated accumulation of damage, extending cellular lifespan. The unique features of the network are assessed by a sensitivity analysis and the simulation outcomes are discussed in relation to available observations of the aging physiome across biological scales.

## Results

### Vicious Cycle Model Development and Simulation

**Figure 2:**
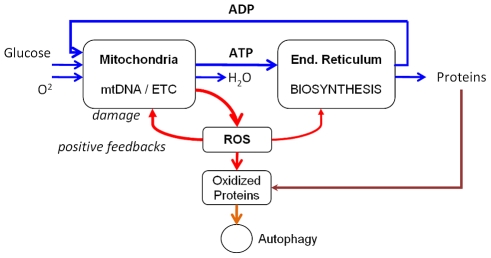
Graph of a positive feedback-loop motif. Several key processes related to biological aging can be described by positive feedback-loop motifs, as shown by this “vicious cycle” model. Metabolic fluxes (marked by blue lines) are initially in homeostasis. Reactive oxygen species (ROS) damage intracellular proteins including mitochondrial structures (red lines). This leads to impairment of ATP generation and biosynthesis, further increasing ROS levels. A portion of oxidized proteins is removed by autophagy, which constitutes a sink in this model.

To demonstrate the role of feedback motifs in the aging process we first develop a simplified aging model in accordance with a positive feedback mechanism or Vicious Cycle (VC), as depicted in Figure 2. One version of the such mechanism has been put forward in a more elaborate form by Bandy and Davison [29] and emphasizes the importance of oxidative damage in aging as a byproduct of oxidative phosphorylation (OXPHOS). Mitochondrial respiration constitutes the main source for various reactive oxygen species (ROS), such as superoxide O_2_^−^, which is converted to oxygen derivatives such as diffusible H_2_O_2_ by superoxide dismutase [30]–[32]. ROS can lead to protein modifications (carbonyl derivatives) and random point mutations in the mitochondrial and nuclear DNA. It has been recognized that mitochondrial damage increases with age, and that this damage to mitochondrial DNA and proteins elevates production of ROS, extensively reviewed elsewhere [33]–[35]. Damage to mitochondrial proteins and DNA closest to the origin of ROS production are most likely, although oxidation of proteins can occur throughout the cell. In the absence of protective mechanisms aberrant mitochondrial proteins and damaging DNA accumulate and a vicious cycle via a positive feedback loop mechanism is initiated. It can be assumed that in this schema oxidative damage to the mitochondria causes energy production (ATP) to decline with time. ROS production through metabolism causes oxidative damage, impairs mitochondrial respiration and, to a lesser extent biosynthesis, reducing homeostasis in metabolic fluxes. Accumulating damage is partly removed in our model by an ATP dependent autophagy (model sink).

The VC graph shown in Figure 2 was subsequently converted into a fuzzy-logic program using the Bionet software (see Materials and Methods). Production of ATP in this model depends on ATP demand, i.e. on the levels of ADP generated through ATP consumption and in the absence of perturbations the model parameters provide homeostasis in metabolic fluxes. The code of the VC model has 8 nodes with 18 processes forming the network topology, as listed in Protocol S1. The reaction parameters, reflecting activities at basal cell-physiological levels, are summarized in Table S1. The initial quantities and concentrations of each node in the VC model are scaled as follows: mitochondrial respiration (MRSP) 0.8, ATP 0.7, ADP 0.7, ATP consumption (ATPconsume) 0.8, protein biosynthesis (ProtBiosynth) 0.8, ROS 0.1 and oxidized proteins (OXPROT) 0.0, i.e. mitochondrial respiration is excellent, ADP and ATP concentrations as well as overall ATP consumption and protein biosynthesis levels are high, and the other parameters are very low. We differentiate in our simulation between overall cellular energy consumption and protein biosynthesis, related to a hierarchy in ATP consumption, which determines that protein biosynthesis declines by 60% when ATP generation is reduced by 30% [36].

**Figure 3:**
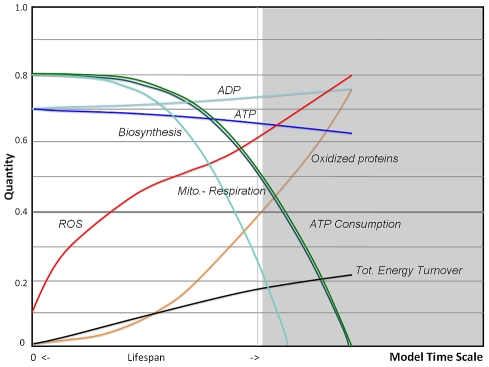
Simulations of a positive feedback-loop motif. A rule-based fuzzy-logic computation shows dynamical changes of age-related alterations in the Vicious Cycle (VC) model as shown in Figure 2. The main characteristic of the simulation outcome is a steep increase in free radicals (ROS) and oxidized proteins due to an amplifying positive feedback, since ROS damages mitochondrial DNA and mitochondrial proteins, impairing the ability to produce ATP, which leads to an increase in ADP levels. Accordingly, mitochondrial respiration and biosynthesis decline exponentially with age. Also provided is the total accumulated energy turnover, which is leveling off with age due to reduced metabolism. High levels of free radical concentrations and protein oxidation are not viable and may lead to cell apoptosis. The end of cellular lifespan is defined here and in all subsequent simulations when oxidized proteins exceeds a 0.4 level, data beyond this point are still shown in shaded areas until ATP consumption reaches zero.

Since in our implementation ROS is a catalyst for the production of ROS by dysfunctional mitochondria, the simulation output of the VC model as shown in Figure 3 reveals an expected steep increase in ROS due to an amplifying positive feedback as the main characteristic of this model. The efficiency of mitochondria to produce ATP becomes strongly compromised with age, but homeostatic regulation reduces the intracellular ATP concentrations only moderately while ADP levels increase accordingly. High levels of ROS are lethal for the cell and dysfunction accumulates rapidly due to insufficient removal of damage by autophagy, therefore we can assume that the ultimate cellular fate of such a mechanism would be apoptosis. While our simulations have been carried out to a point where mitochondrial respiration reach a zero level, the limit of viability or fitness of a cell is expected to occur earlier and is compounded by mechanisms in the apoptotic pathway [37], [38]. Here, we define the end of cellular lifespan when oxidized proteins (OXPROT) exceed a level of 0.4 and we use this ad-hoc criterion to compare simulations outcomes of different model implementations. Oxidized proteins, including lipofuscin, carbonyl derivatives and advanced glycation end products (AGE), have been found to be an excellent marker of lifespan prediction in cells and model organisms [39]–[41].

### Critique of the Vicious Cycle Model

The vicious cycle theory as it relates to the mitochondrial mutational theory of aging has received both criticism and extensions after its introduction. Here we first review related aspects to provide a basis to extend our model in complexity, as shown in Figure 1. One line of discussion refers to mitochondrial biogenesis, suggesting preferential survival of dysfunctional mitochondria [42] and expansion of mutations by positive feedback loops related to decline in ATP levels [43], [44]. On the other hand, it was not confirmed that mtDNA mutations spread exponentially within a given cell by clonal expansion, rather the prevalence of single or a few mtDNA point mutations has been observed [45], [46]. These findings have to be seen in context of many observations that mitochondrial function indeed declines significantly with age, as reviewed elsewhere [33], [47], and that experimentally induced oxidative stress in mitochondria increases ROS levels, but not necessarily in an exponential fashion [48].

However, the hypothesis that aging is only a process of random damage unnoticed by mechanisms of cell defense system is flawed in view of recent experimental findings, including the aforementioned activation of transcriptional responses. Adaptive behavior to mitochondrial dysfunction has been termed retrograde response, a mitochondrial-to-nucleus cross talk first described in yeast as a rheostat mechanism involving retrograde response proteins [49], [50]. For eukaryotes, the transcription factor NF-κB may have a similar role in sensing mitochondrial stress with respect to perturbations in concentrations of second messengers [51], [52].

On this basis we extend the Vicious Cycle (VC) model towards an Adaptive Response (AR) model characterized by negative feedback loops, driven by NF-κB, which counteract the generation of oxidative damage and prevent excessive ROS generation. The role of NF-κB in aging has been first identified as causal for downregulation of the androgen receptor gene in aged liver [53], it was subsequently found at elevated levels in many other tissues [54]–[56] and more recently we reported higher-DNA binding activity of NF-κB in quiescent, non-proliferating fibroblast cell cultures from older donors [57], representing closely the physiological state of the majority of cells *in-vivo*. In contrast to the activation of NF-κB through classical pathways such as TNF-α, primary activation in aging is a constitutive and cell-intrinsic “atypical” mechanism [24], contributing to a low-grade inflammatory state in aged organisms [58], [59]. In consideration of a diverse range of activating mechanisms of NF-κB, the AR model considers not only increasing ROS levels caused by dysfunctional mitochondria, but also accumulated oxidative damage, observed as lipofuscin, mediating the stress response [39], [60], [61]. A related positive feedback loop considers the activity of inflammatory markers such as cytokines regulated by NF-κB, which activate the nonphagocytic NADPH-oxidase system [62], [63], contributing to higher levels of free radicals in older cells.

Notably, other types of feedback have also been identified in aging. Lower protein output in aged cells decreases overall protein turnover, so that the portion of damaged proteins accumulates more quickly, a process which may be further enhanced by less efficient ubiquitination and degradation [64], [65]. The kinase mTOR has been identified as an important key regulator of autophagy [28], while mTOR's complex 1 (mTORC1) is also an important regulator of biosynthesis, negatively regulated by available ATP concentrations involving upstream adenosine monophosphate-activated protein kinase (AMPK) [66]. The Vicious Cycle model shows lower ATP and higher ADP levels indicating insufficient ATP supply due to damaged mitochondria, which is in agreement with observations that ATP levels in some tissues decrease with age and cause constitutively increased AMPK levels [67]–[69]. Modulation of mTOR levels may be tissue specific and pronounced in cells with transiently varying energy demand (muscle, neurons). Consequently mTOR activity, if suppressed in aging [70], [71], would promote autophagy as well as metabolic adjustments and adaptation of mitochondrial capacity in a feed-forward fashion [27]. However, it has also been found that higher levels of ROS can activate mTOR [72], which is physiologically relevant for responses to nutrition, but counteracting the beneficial suppression if chronically activated in aging. We therefore incorporated both NF-κB and mTOR as important stress sensors and metabolic mediators into our model, as described in detail below.

### Adaptive Response Model Development and Simulation

**Figure 4:**
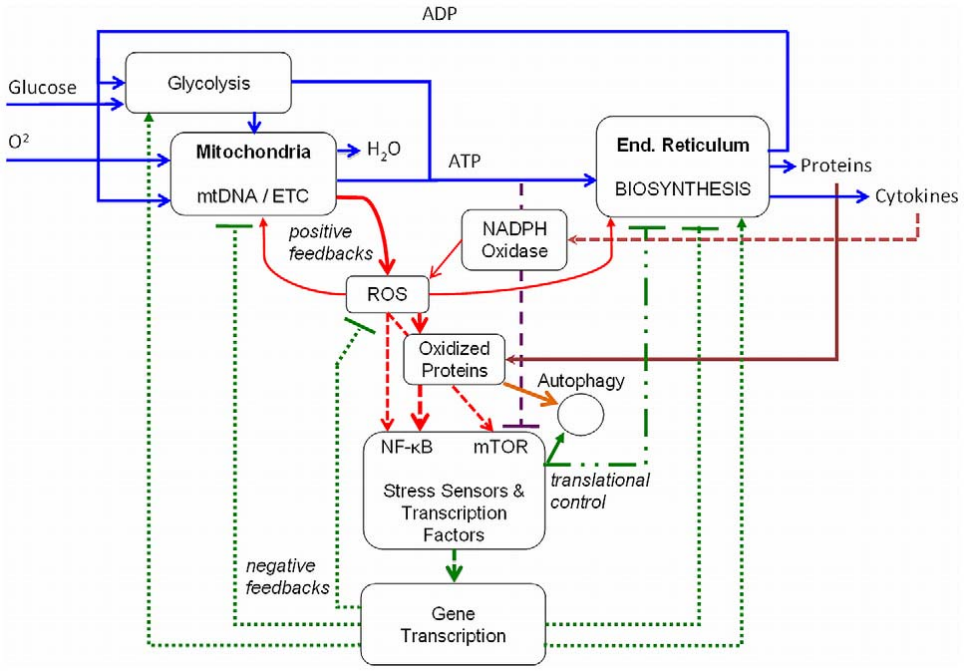
Circuitry of the Adaptive Response model. The Adaptive Response (AR) model represents an interaction network topology with both positive-destructive and negative-protective feedback mechanisms co-existing in cellular aging. Dysfunctional mitochondria and a decrease in protein turnover are positive feedbacks and contribute to the accumulation of oxidized proteins. Increased levels of ROS and oxidized proteins activate the redox-sensitive stress response transcription factor NF-κB, while declining ATP levels inhibit the energy sensor mTOR, which supports negative feedbacks through changes in transcription and translation (dotted green lines indicate flow of information and arrow endstyles the suggested function in aging). This includes downregulation of protein biosynthesis and genes coding for mitochondrial proteins. In addition, the activity of scavengers and autophagy is enhanced. A compensatory mechanism to mitochondrial dysfunction is upregulation of aerobic glycolysis. Secondary positive feedback-loops incorporate the production of cytokines as a byproduct of the cell-autonomous response of NF-κB, activating the NADPH oxidase system in an autocrine fashion, as well as reduced protein turnover rates. The beneficial role of mTOR inhibition in aging may be blunted by high ROS concentrations (see Results for details).

Central to the adaptive-response model is the presence of stress-response elements NF-κB and mTOR that alter transcription and translation for antioxidants, biosynthesis and mitochondrial function, as shown by the interaction map in Figure 4. Initial conditions in the AR model and basal rates, including rates of oxidative damage, were adopted from the VC model to allow direct comparison of the performance of both models (Table S1). Initial condition for NF-κB activity was set to 0.07 and mTOR activity (representing pmTOR) to 0.4. The model also uses the same basic rules for generation of ROS, buildup of OXPROT damage and inhibition of mitochondrial respiration. However, the AR model has additional nodes (n = 10) and processes (n = 36) with rules defined for 71 combinations (see Protocol S1), including mechanisms for NF-κB and mTOR activation and three negative feedback loops reducing metabolic respiration, biosynthesis and improving ROS scavenging, as shown in Figure 4. Further included are a positive feedback loop considering increase in the relative portion of oxidized proteins through reduced turnover rates of newly formed proteins, a compensatory mechanism stimulating glycolysis, and a secondary autocrine loop activating the NADPH oxidase system. We reference the relevant literature as we describe the implementation of these feedback mechanisms.

**Figure 5:**
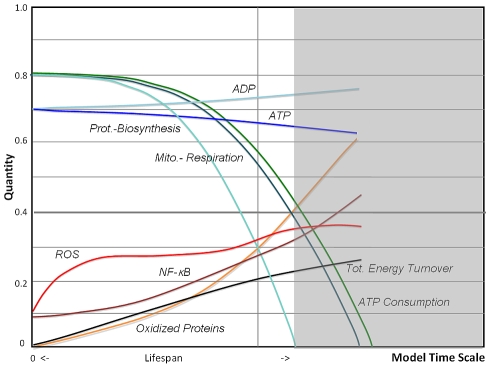
Fuzzy-logic simulation of a model including NF-κB. In this simulation the VC-model is extended towards an adaptive response (AR) model by introducing the NF-κB pathway that protectively upregulates ROS scavengers and downregulates mitochondrial function. Compensatory upregulation of aerobic glycolysis diverts ATP consumption from mitochondrial respiration. Model lifespan is only slightly extended and the model still shows an accelerated decline.

The first negative feedback loop added to the VC model concerned the activity of NF-κB, but with only minor involvement in metabolic activities. This transcription factor is known to activate a cellular survival response that includes scavenging of free radicals. For instance, MnSOD has been shown to be increased with age and is NF-κB dependent [73]. Notably, NF-κB is co-localized with mitochondria and is a potential regulator of mitochondrial respiration [74], acting as a mitochondrial inhibitor in our model. Although NF-κB is not known to have direct target genes related to metabolism, as determined during TNFα stimulation [75], it interacts with other transcription factors that have such target genes, including c-MYC [76], [77] with a role in glycolysis, as well as YY1 with a role in mitochondrial biogenesis [78], and both mechanisms were incorporated. The simulation output is shown in Figure 5. While concentrations of ROS and oxidized proteins accumulate more slowly, the system still shows an accelerated decline in basic functions. In a second step the activity of the kinase mTOR was added to the model, downregulated by low ATP concentrations, but activated by high levels of ROS [72]. The resulting feedback loops involve translationally regulated targets in the endoplasmic reticulum modulating biosynthesis [79]–[81] and the prevailing inhibition in aging is further supported by lower levels of ribosomal transcripts in different tissues types as indicated by gene expression studies [80].

Not only a decrease in functional mitochondrial capacity [82], [83], but also downregulated gene transcripts of mitochondrial genes coded in nuclear DNA have been reported [80], [81], [84]–[86]. Related lower levels of NADH dehydrogenases, alterations in the TCA cycle, electron transport chain and mitochondrial membrane potential, contribute to reduce respiration and ATP production, which can occur before any damage is observed [87]. This is initially counterintuitive, since we may expect an increase in biogenesis in response to dysfunction of damaged mitochondria, which is only partially observed at lower mTOR activity [88]. However, it fits the notion that an active downregulation of respiration, rather than damage only, contributes to the observed progressive decline in mitochondrial energetics. Accordingly, active downregulation of mitochondria mediated by mTOR [89], complementing the role of NF-κB, was implemented as a further negative feedback into the model. As a potentially compensatory mechanism we incorporated anaerobic glycolysis into our model (see Figure 4). The contributing rate of glycolysis towards ATP production in resting cells is very low, but increases substantially when cells proliferate [90]. However, proliferating cells were not the basis for our model assumptions, therefore basal glycolysis rates are not explicitly shown unless the rates were increased, which is known to occur in aging [91], [92] and is mediated by NF-κB and mTOR [27]. Finally, mTOR activity negatively regulates autophagy [28], which was set to a low rate in the VC model. Secondary positive feedback loops in the model include reduced protein turnover and activation of ROS by the nonphagocytic NADPH-oxidase system [62], initiated through an autocrine effect due to the secretion of cytokines regulated by NF-κB. At this stage parameters for the interaction of NF-κB and mTOR within the network topology were fine-tuned using available data (see Materials and Methods).

**Figure 6 pcbi-1000820-g006:**
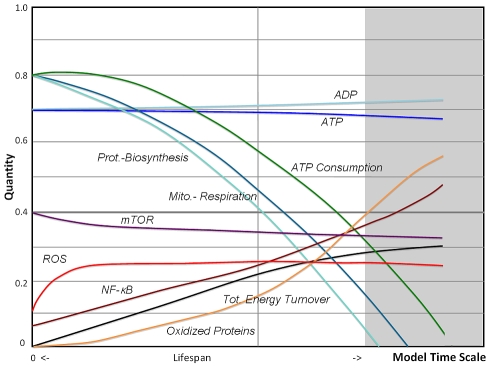
Fuzzy-logic simulation of the Adaptive Response model. In this simulation of the complete AR-model (see graph in Figure 4) concentration of reactive molecules stay constant at low levels and oxidized proteins accumulate slower increasing lifespan, different to the model predictions shown in Figures 3 and 5. In this setting, oxidized proteins become the main mechanism for activation of the stress response sensor NF-κB. Low ATP values decrease mTOR, downregulate ribosomal functions, but enhance autophagy as protecting mechanisms. The underlying alterations in gene transcription and translation decrease mitochondrial respiration but upregulate aerobic glycolysis, which becomes a major contributing factor to energy supply towards the end of lifespan. The overall accumulated energy turnover is higher compared to all other models. The AR model demonstrates an earlier onset and more linear rates of decline for energy related parameters if compared to the aging phenotype predicted by the Vicious Cycle model.

Simulation outcome of the AR model is shown in Figure 6 and reveals a substantial improvement in lifespan along with a reduced biosynthesis and improved autophagy, when compared to Figure 5. At the same time glycolysis is substantially enhanced and the lines for mitochondrial respiration and ATP consumption diverge. The net effect on the overall shape of metabolic decline is an inward bending of the exponential characteristics towards a more linear decline, consistent with findings of mitochondrial functions in a longitudinal study [83]. In contrast to the VC-model, the limiting factor for cell survival in the AR model is not only found in high levels of oxidative stress and damage, but also in low energy states which may be insufficient to support cell survival and higher-level functions.

### Predictions of Feedback-Loop Inhibitions

Having established a generic network model capable to predict the role of stress responses on the aging process, we asked whether this model would be able to correctly predict the outcome of experimental interventions performed on the stress sensors NF-κB and mTOR. Since the long-term effects of such interventions cannot be simulated in cell cultures, such interventions are usually performed in model organisms and the effect on mean lifespan is determined.

**Figure 7 pcbi-1000820-g007:**
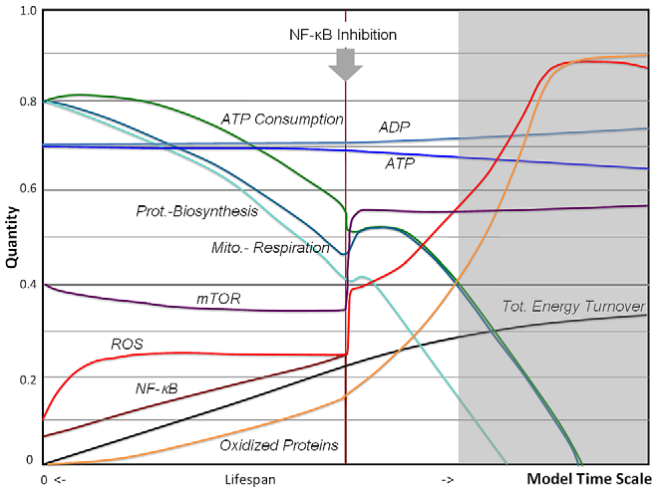
Simulation of NF-κB blockade. In this version of the AR model NF-κB is inhibited from a mid-point time on. The scavenging role of NF-κB and its role in mitochondrial regulation, along with increased biosynthesis by ROS activation of mTOR, cause an instant improvement of metabolic functions moving the cell to a “younger state”, consistent with NF-kB blockade experiments. However, the model prediction after this point in time has the characteristic of a vicious cycle with accelerated decline not improving lifespan.

In assessing the role of the stress sensor NF-κB, Figure 7 shows the simulated outcome of an inhibitory experiment in which the concentration of NF-κB is reduced during midlife. The model reveals an instant improvement of mitochondrial metabolic and biosynthesis functions. This inhibition predicts increase in levels of ROS, by canceling of the role of NF-κB in ROS scavenging and mediation of mitochondrial respiration. At the same time increased ROS levels activate mTOR and increase biosynthesis, which accelerate the aging process [93]. Since the model converts to that of the vicious cycle system for the remainder of the simulation, no overall increase lifespan is noticed. However, the effect on lifespan will depend on the weighting of ROS contributed by dysfunctional mitochondria against ROS contributed by the NADPH system, which could be experimentally determined by an NF-κB blockade experiment. There is early evidence that blockade of the NF-κB pathway in tissues may indeed return cells transiently to a biologically “younger” states as demonstrated by restoration of the transcriptional profile and histo-morphology in skin tissues [54]. However, the predicted increase in oxidative stress and related damage will not increase lifespan according to our simulation, consistent with premature aging observed in NF-κB p50 knockout mice [94], and the change in redox state may further account for an observed predisposition of the epidermis to neoplasias in NF-κB inhibitory experiments [95]. Nonetheless, these simulation demonstrate the role of regulation and plasticity in the aging process, as suggested previously [96], since cellular function and potentially related epigenetic effects can be restored to the extent that function is not impaired by damage. In contrast, the vicious cycle model does not provide access points to change cellular behavior because dysfunction stems from accumulated damage and cannot be reversed.

**Figure 8:**
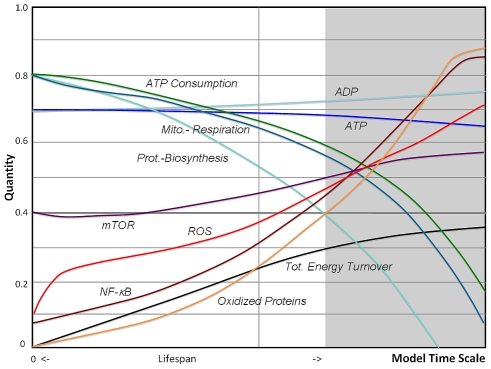
Simulation of decreased mTOR sensitivity. In this version of the AR model mTOR sensitivity to lower ATP levels is decreased by 20% and lifespan is compared to the simulation in Figure 6. An initial decline in mTOR becomes reversed by increasing ROS levels, enhanced by mTOR mediated activation of biosynthesis and mitochondrial activities. This is an example of “unsuccessful aging”, demonstrating the critical role of mTOR in the regulation of the aging process.

Next we reduced mTOR sensitivity to low ATP levels by 20% (process MTOR_ATP) and derived a prediction shown in Figure 8. While mTOR levels initially fell slightly below the initial value, they then increase throughout the simulation and a strong biosynthesis elevates free radical levels, which are even higher if compared to the sole activity of the NF-κB model shown in Figure 5. This is an example of “unsuccessful” aging and high mTOR activities are frequently discussed in relation with a senescent phenotype and age-related diseases [97]–[100]. Whether mTOR downregulation through genome damage involving the ATM pathway plays a contributing role in aging will need clarification [101]. But we begin to see how rapamycin, caloric restriction and Sirtuins, all known to suppress the mTOR pathway, can extend lifespan in model organisms such as yeast [102], *C. elegans* [103] and mice [104]. The dependency of the degree of inhibition on lifespan is examined below.

### Sensitivity Analysis

Because the complicated nature of network models it becomes necessary to analyze their behavior quantitatively on a broader scale [105]–[107]. Sensitivity analysis is a preferred way to determine the contribution of individual network components onto the overall behavior of the network. First we tested reaction rates of the AR model (details are provided in the Text S1), initially increasing the rate of each process by 5%. Lifespan fitness parameters included ROS exceeding a threshold level of 0.4, oxidized proteins exceeding a level of 0.4, or when ATP consumption fell below a level of 0.4. A change in the Sensitivity Objective Function (SOF) [108], here defined as the ratio of the percent change in lifespan with the percent change in the selected reaction rate, was seen as significant if |SOF| >2.5. The most significant 13 reactions from this analysis were also changed by −5% and +/−20%. Reactions initially tuned to provide homeostasis in metabolic fluxes (including MRSP_deactivation, Biosynth_consumed, MRSP_ADP and ATP_used) produced major changes when their reaction rate was increased. The remaining reactions had less impact except reactions involving mTOR (e.g. MTOR_ATP). No linearity with the degree of rate change on lifespan was observed, since fitness parameters can shift in opposing directions when individual rates are changed. A linear regression analysis did not show a statistically significant relationship between the magnitude of the reaction rates and their percent changes (see Text S1).

**Figure 9:**
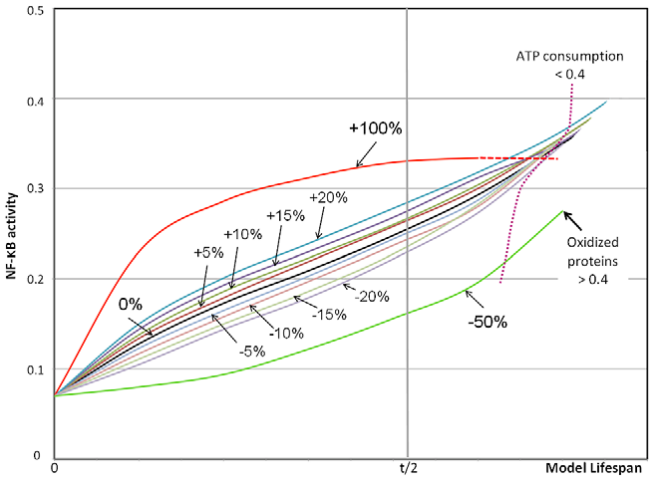
Sensitivity analysis of NF-κB. In this analysis the sensitivity of NF-κB is modified between +/−5 and +/−20%, and by +100% and −50%. The NF-κB activity is plotted over the course of lifespan as recorded by simulation runs compared to a baseline (black) from model predictions shown in Figure 6. End of lifespan is reached when the level of oxidized proteins reaches 0.4 as indicated. A second fitness criterion indicates ATP consumption levels at a 0.4 threshold (dotted magenta line). Substantial differences in NF-κB activity are observed around midlife but converge later without affecting overall lifespan. Substantial reduction in lifespan is only seen at very low NF-κB values along with damage related low levels of ATP consumption.

**Figure 10:**
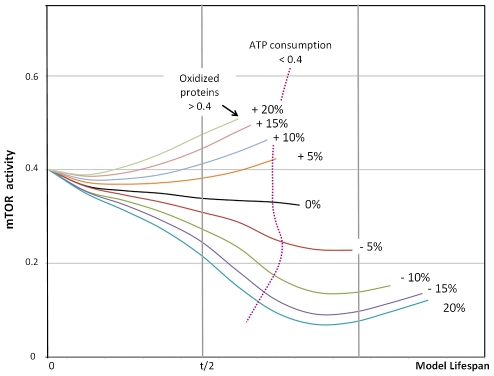
Sensitivity analysis of mTOR. Alteration in mTOR sensitivity to low ATP levels introduced in steps of +/−5% have a significant effect on lifespan, as indicated by the length of simulations runs carried out until oxidized protein concentrations reach a critical level. Changes do not increase in proportion to higher degrees of perturbations. It is noted that mTOR activities at positive changes from the baseline (black line) can initially decline, but increase later. Stronger inhibition of mTOR slows accumulation of oxidized proteins, but reduces other fitness parameters such as ATP consumption (dotted magenta line) that may negatively impact physiological functions.

To further elucidate the underlying mechanisms we performed another sensitivity analysis of the stress sensors NF-κB and mTOR and examined their behavior over elapsed time. Each activating parameter (sensitivity of NF-κB to be activated by OXPROT and ROS, and mTOR to be activated by low ATP levels) was modified in steps of +/−5% over a range of +/−20%. The results for NF-κB are provided in Figure 9 and for mTOR in Figure 10, in which the activity of both species are shown. Simulations were stopped when a fitness criterion (oxidized proteins >0.4) was reached. The results show that the behavior of NF-κB and mTOR differ. NF-κB values initially divert and are most pronounced at midlife but converge in old age, therefore the overall impact on lifespan is minor, consistent with results from the SOF analysis. We recall that NF-κB in our simulation is activated by both ROS levels and accumulated damage, it is involved in mitochondrial activity and scavenging, but also increases ROS levels by an autocrine loop through involvement of the NADPH oxidase system when activated. These complex interactions and responses at low sensitivity of NF-κB let oxidative proteins accumulate more rapidly, but only if a major reduction is introduced (-50%) then a substantial reduction in lifespan is seen (Figure 9). A higher sensitivity of NF-κB causes a disproportional higher production of ROS through the NADPH oxidase system accompanied by a strong downregulation in mitochondrial activity reducing ATP availability. Such responses to high NF-κB activity may suggest a mechanism for increased human frailty when exposed to inflammatory episodes [109], [110].

In contrast, mTOR activity shows a more pronounced pattern of variability at small perturbations along with a substantial change in lifespan increasing from low to high sensitivity of ATP concentrations (Figure 10). The sensitivity to changes is pronounced around the initially set values, but perturbations over 10% do not lead to a proportional change. Low mTOR values demonstrate the beneficial effect on lifespan, limiting accumulation of oxidized proteins and damaged mitochondria. However, other fitness parameters such as ATP consumption become impaired as indicated in Figure 10, which shows physiological limitations. An interesting observation is the possibility for initially suppressed, but later increased mTOR activity, which will await experimental validation.

In summary, our generic model can make qualitative predictions of interventions performed at stress sensors on lifespan extension that involve a complex interplay of metabolism and damage progression. Such computational simulations support identification of balances between the different demands of a cell or organism to keep relevant fitness criteria at optimal levels in order to promote healthspan.

## Discussion

Many processes in biology, including those related to aging, are not well enough quantified to make rigid mechanistic-mathematical modeling applicable, although they are qualitatively understood by their underlying rules. In consideration of the opportunities presented by the utility of hybrid-intelligent tools we introduced the development of a cell systems model of aging, derived from a manually curated integration of available experimental evidence. In the current absence of precise experimental measures of all parameters involved, the choice of a fuzzy logic approach allowed analysis of logical interactions and semi-quantitative simulation for phenotypical predictions. For application to the biology of aging, the rule-based approach has advantages over other logic-based methods, such as Boolean or Baysian networks. The framework allowed us to encode components in six intermediate values and relationships in close expressions of natural language, while in Boolean networks properties are encoded as either “0” or “1” and logical interactions are restricted to “AND”, “OR” and “NOT” gates [111]. However, most nodes in the aging network, such as mitochondrial respiration or stress sensors, are not in an “on” or “off” state, rather change continuously over time. The model uses defined membership functions and interactions, different than Bayesian networks, which assign probabilities whether certain interactions or reactions take place or not to handle uncertainty in signaling networks [112]–[114]. Therefore, we argue that the approach used here is an appropriate choice to generate fairly realistic predictions, which is in accordance with experiences of rule-based and FL systems in other biosimulation areas [10], [11], [115]–[117].

The constructed graphs use feedback-loop motifs to model key cellular processes and their alteration with age functionally. The models are semi-quantitative and coarse-grain in nature, but demonstrate logical dependencies among metabolism, damage and signaling. The simulations support a conception that the aging phenotype is not only dictated by irreversible accumulated damage but may have substantial regulatory and protective components. While it has been shown that specific, experimentally isolated processes related to aging can be identified and modeled mechanistically [118]–[120], our model predictions are different from an earlier approach that combined several submodels for mitochondrial reactive oxygen species production, aberrant proteins, free radicals and scavengers, but did not include mechanisms of stress responses and predicted significant changes only in old age [121]. Furthermore, our approach goes beyond generalizing mathematical descriptions rooted in systems theory [122], [123] or global protein-protein interaction network analysis [124], but rather connects to specific tractable molecular mechanisms.

Our model mostly relies on data from human skin fibroblasts in longitudinal studies (see references in Materials and Methods) and not all parameters are currently available experimentally to fully scale, detail or optimize the model. Model scaling has to be performed with respect to time, amplitude and rates and similarity of the model outcome with experimental data can be determined by a regression analysis [125] when such data become available.

### Model Predictions of Cellular Aging

It has been pointed out earlier that aging can be understood in terms of lack of a total quality control [126], which would be uneconomical for the cell to entertain since it requires a complex and energetically expensive control system. Therefore damage is slowly accumulating but in accordance with our current understanding the cell activates adaptive mechanisms to contain the rate of accumulating damage extending its survival. In our model the generation of reactive oxygen species as a byproduct of mitochondrial respiration, and imperfections that concern the removal of free radicals and oxidative damage, is the proximate cause of aging. The role of mitochondrial ROS in aging has been widely discussed since its introduction [127], and while ROS production in older cells may be increased under certain conditions, this may not be the case in basal metabolism. This fits well with the critical role of ROS in biological species and its function as a messenger molecule [128], [129], a property which may be lost if ROS levels exceed the normal regulative range. The AR model predicts that ROS through downregulation of mitochondrial respiration, known to be related to lower membrane potential [130], stays in a more narrow range and is contained (Figure 3 vs Figures 5 and 6).

Inhibition of mitochondrial respiration as a protective mechanism bends metabolically related curves towards a more linear characteristic and mitochondria would not require the mutational load or damage required in the vicious cycle theory. Regulatory mechanisms for mitochondrial function may be specific for each tissue, and other suggested mechanisms such as uncoupling proteins to reduce ATP synthesis [131] can be seamlessly integrated into this concept. Our predictions are consistent with findings from the mitochondrial mutator mouse featuring a mutation in the proofreading mitochondrial DNA polymerase γ, which had been used to study the influence of mitochondrial dysfunction on aging [132], [133]. The results of these particular studies had shown no change in free radical levels of prematurely aging mice. In reference to our model, we suggest that an earlier protective downregulation of mitochondrial respiration and metabolism plays a role in the accelerated aging of the mutator mice, in prevention of high levels of oxidative stress, potentially increasing the rate of apoptosis if compared to the wildtype.

According to our model, NF-κB and mTOR activities become intrinsically altered through accumulating damage, modest mitochondrial dysfunction and less efficient energy metabolism. There is early evidence that both pathways cross-talk [134], [135], but this relationship is not well understood. While both stress sensors protectively remodel metabolic processes and extend lifespan, they also enforce this process by steering the cell into a lower energetic state with reduced protein turnover. The beneficial role of mTOR at low ATP concentrations could be compensated by insufficient ROS elimination, suggesting a distinct mechanism for the frequently discussed insufficiency of autophagy in some age related diseases. The mediating role of the multichannel stress sensors NF-κB and mTOR as discussed here challenges the current view that aging happens outside evolutionary shaped mechanisms. Rather, diverse forms of stresses are canalized into stress-response pathways, which have evolved to protect against extrinsic environmental insults, nutritional shortages and infections, causing predictable cellular alterations. Mechanisms which shape the response of these pathways include exogenous factors such as chronic inflammation [109], epigenetic mechanisms and genomic stability [136], [137], [138], as well as the potential role of genetic “buffering” factors including those involved in inflammation, such as adiponectins [139]. Additional stress response pathways discussed in relation to aging such as p53 [140], JNK [141], FOXO [142], Cap'n'collar transcription [143] and heat shock factors [144], [145], as well as the effect of enrichment of stress resistance genes in *C. elegans* [146], provide opportunities to extend model detail. A sensitivity analysis as applied above should support identification of the most influential components, complementing experimental knockout or pharmacological intervention studies.

### Optimality of Aging and the Physiome

A theoretical optimum must exist for each cell in relation to its genetic makeup [147]–[149], and its metabolic tasks within an organism of given size [150], to live long. According to our simulations and available experimental evidence, aging cell phenotypes *in-vivo* may not be far away from an optimal path. However, age-related pathologies and cellular senescence mark loss of optimality. Therefore, inhibition of pathways that promote metabolism such as mTOR may extend healthspan [151]–[154], but can reduce fitness in other areas as shown by our sensitivity analysis (Figure 10). Consistent with this, a considerable slowing of behavioral functions has been observed in model organisms treated with inhibitors of mitochondrial respiration [155] and lifespan extension was limited when pharmacological inhibition of metabolic pathways was additionally combined with caloric restriction [103]. Furthermore, we hypothesize that the postulated optimum may be close to a linear decline in energy metabolism, which, according to our model, seems to be related to the requirement of keeping second messengers at low levels to maintain an environment in which signaling tasks can be executed [128], [129].

Although one can expect a considerable degree of complexity when extrapolating from molecular mechanisms to the levels of tissue, organisms or populations, some basic principles appear to make imprints across levels of biological organization. If cellular aging were driven by an exponential increase in dysfunction as predicted by a vicious cycle model, then there should be a noticeable accelerated dysfunction on higher levels of biological organization, from organs to populations. Physiological observations and population models belie this assertion.

First, a linear decline of many physiological parameters in humans can be observed, as initially reported by Nathan Shock [156], [157]. Later, in a comprehensive review of loss rates of 445 physiological parameters composed from 469 studies, a linear fit with an average loss of 0.65% per year was revealed [158]. It would be difficult to explain how an early onset and linear loss rates would be compatible with an exponential decline on the cellular level as predicted by the vicious cycle theory and the first stages of our model implementation. For this reason it can be proposed that a linear characteristic of so many different physiological processes is driven by a linearity in metabolic remodeling on the cellular level as demonstrated by the AR-model (see also Figure 6), which was assembled to show an average behavior of somatic cells in human tissues with low mitotic index representing a major portion of the body mass.

Secondly, linearity in decline of cellular and physiological functions may also shape population statistics. In humans and model organisms a plateau in mortality rates has been observed at high ages [159]. In an attempt to describe this behavior mathematically, Weitz and Fraser [160] suggested to interpret individual viability as a function of constant drift, resembling loss of function, combined with a stochastic Gaussian fluctuation, also observed in model organisms [161]. A proper weighting of loss rates and random components in cohorts generates a plateau in mortality rates as observed in the human population. This surprisingly demonstrates that despite the biological complexity involved in the mechanism of aging, a rather simplistic property of linearity in decline may dominate the aging physiome across levels of biological organization.

### Conclusions

The overall thrust of computer modeling in aging is to decipher and predict the role of molecular complexities and dynamics underlying the aging phenotype. While experiments and perturbations on the cellular level provide crucial insights into actual network states, these experiments are limited to predict long-term mechanisms and behaviors. Therefore, the ability to perform computer simulations over entire lifetimes provides a crucial aid to investigate rapidly and repeatedly the effect of molecular mechanisms on long-term cellular behaviors. The generic model as introduced here provides a critical complexity to make useful qualitative predictions but is only a first step into this direction. It will require an in-depth consideration of cell cycles, cell end states, tissue compartmentalization, different physiological functions and endocrine regulation, before such predictions can be fully merged with lifespan measures in model organisms. As mechanisms will be reflected at increasing level of detail drawn from more integrated experimental setups, such models are expected to become valuable for hypothesis generation, testing and identification of critical molecular targets and mechanisms underlying aging and age-related chronic diseases.

## Materials and Methods

The cell network models presented here are based on a computational fuzzy logic (FL) framework. The calculations for interactions between nodes are similar to functional Petri nets [162], with processes replaced by fuzzy logic inference instead of sets of differential equations. In traditional systems modeling, each node of a network represents the quantity of some specific protein or gene. In fuzzy logic modeling, we can use a node to represent broader entities, such as mitochondrial respiration, the demand for ATP, or other abstract quantities. Fuzzy logic modeling is used widely in complex engineering applications [163]–[165], and appears to be particularly suitable since cellular biodynamics may be considered a complex control system.

We used a computational framework termed Bionet for our simulations [10] (downloadable at https://simtk.org/home/bionet/). According to the concept of fuzzy logic the range of each node was normalized from zero to unity and within this range six possible states were defined, which is an advantage over discrete and dynamic Boolean models [166] for our application. The ability to provide intermediate values, rather than binary parameters used in binary-logic models, allows us to draw conclusions about slowly progressing state changes. Bionet defines four possible types of processes or reactants: elements which are produced (product) or used up (substrate), and with respect to their rates these processes can be either accelerated (activator) or delayed (inhibitor). The reactions respond to inputs according to default rules and for each process where the rate constants and the role of all processes are defined before the model is executed (Protocol S1). Key processes and phenotypical descriptors (nodes) included ROS (the level of reactive oxygen species), OXPROT (oxidative protein damage), MRSP (respiration of the mitochondria representing functional activity of this organelle), ATP (the amount of ATP produced by the mitochondria), ProtBiosynth (the biosynthesis of proteins by the endoplasmic reticulum), ADP (the amount of ADP released after biosynthesis), NFkB (stress sensor and transcription factor NF-κB) and mTOR (kinase acting as ATP sensor and translational metabolic regulator).

Connectivity of the overall network topology was defined by activating factors in production and decay, based on fuzzy-rule-based inference. For instance, the process of ATP generation was coded to be accelerated by ADP related mitochondrial respiration by defining a reaction process and rate “Reaction MRSP_ATP 0.8”, and in the Bionet syntax “pro ATP 5 5 5 5 5 0”, and “act MRSP 0 1 2 3 4 5”. This expresses in terms of natural language that “regardless of ATP concentration, the amount of mitochondrial activity determines ATP production, until the upper limit of the scale is reached (saturation)”. As another example, the default value of mTOR was set to 0.4, centered at the third of the available six fuzzy states. Functions depending on mTOR reflect this property. For instance, the rate of autophagy was determined by the reaction Autophagy_MTOR with the formulations “sub OXPROT 1 2 3 4 5 5” and “act MTOR 5 2 1 0 0 0”. Hereby the rate of autophagy became dependent upon the concentration of oxidized proteins and is more strongly promoted when mTOR reached levels below 0.4.

Mathematically, Bionet builds on the standard additive model (SAM), a common formulation of a fuzzy logic system. SAM is a universal approximator and can approximate any nonlinear function as precisely as desired, given appropriate data [167]. Once the real numbers are transformed into fuzzy or linguistic variables first, the outputs of the system are computed by the inference system, and then the fuzzy output variables are transformed into real number outputs. The membership functions used in Bionet to transform between real numbers and fuzzy variables are linear triangular functions using the aforementioned six fuzzy variables on the real interval from 0 to 1 (which can trivially be generalized to any real interval). The triangular membership functions, to which linguistic names may be assigned, overlap such that every real number in the interval belongs to either one or two fuzzy sets. Real numbers that fall exactly on the centroid *C_i_* of a membership interval belong 100% to that variable. Those that do not fall on a centroid belong fractionally to two fuzzy sets. Thus, mapping from a real number *R* to fuzzy variables is accomplished uniquely by:
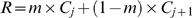
(1)with m ∈ [0.0,1.0].

The fuzzy inference engine, which is the universal function approximator, maps fuzzy inputs to fuzzy outputs. The inputs are assigned fractional weights, as in equation 1, and the same fractional weights are applied to the output fuzzy variables, which are then mapped back to real number outputs in the same manner. Details of the standard additive model (SAM) are provided in [10].

One underlying goal of our study was to evolve models that would allow comparison of their predictions. Initially we started with a system that provides homeostasis in metabolic fluxes following the classic concept of dependencies between mitochondrial respiration and ATP consumption (see Figure 1). This system was extended to include process of damage in terms of a positive feedback loop. All parameters used in the VC model were subsequently carried over to the AR model, to demonstrate the influence of negative feedback loops.

For the final model, rate constants were manually adjusted to provide relationships to available experimental data, primary from studies of human pre-senescent fibroblasts. Fibroblasts from cross-sectional or longitudinal studies are one of the best-investigated cell systems in aging. Experimental results considered here include: a) the decline in mitochondrial respiration, which show a linear decline from donors aged 20 to 94 years in a longitudinal study [83], b) exponential increase with age in oxidized protein content [168], c) reduction of ATP levels [38] and d) increasing levels of NF-κB, which demonstrates a low-grade inflammation increasing with donor age, in contrast to high values found in acute inflammation [57]. Additional relationships considered stem from investigations with other mammalian cell types, such as hierarchy of ATP-consuming processes [36], the rate of ATP production by aerobic glycolysis to that of OXPHOS [90], alterations of the ATP/ADP ratio [67], [71] and increase of glycolysis with age [91], [92]. Within the given time window (model time scale runs from 0 to 40, as displayed in Figures 3 and 5–8) 10000 iterations were carried out. The Bionet output file was imported into MS Excel and the data was averaged before graphs were plotted.

## Supporting Information

Protocol S1 List of the VC and AR model Bionet software.
(0.07 MB PDF)

Table S1 List of processes and rate constants.
(0.08 MB PDF)

Text S1 Description of the sensitivity analysis.
(0.16 MB PDF)
