# Supplementary material for: Rule-Based Cell Systems Model of Aging using Feedback Loop Motifs Mediated by Stress Responses
Source: PLoS Comput Biol. 2010 Jun 17;6(6):e1000820. doi: 10.1371/journal.pcbi.1000820 (PMC2887462; doi:10.1371/journal.pcbi.1000820)
Supplement: Protocol S1 — List of the VC and AR model Bionet software. (0.07 MB PDF) [file pcbi.1000820.s001.pdf]

Supplement Protocol S1 – Bionet Program Listings

Vicious Cycle (VC) Model

```
#  Hypothesis Vicious Cycle
#
#  Jan 5, 2010
#
nFuzzyStates 6

starttime 0.0
endtime 40.0
ntimesteps 10000

PopulationSize 1
nGenerations 1
mutationRate 0.05

Exp

# Define variables to be plotted
Plot ROS
# Level of Free Radicals
Plot OxProt
# Level of oxidized proteins
Plot MRSP
# Mitochondrial Respiration
Plot ATP
# ATP levels
Plot ADP
# ADP levels
Plot Energy_tot
# Plot total energy consumed
Plot ATPconsume
# Plot total energy consumed
Plot ProtBiosynth
# Biosynthesis Activity levels
#
# Define nodes, start with initial condition
# High ATP and Biosynthesis, ROS and NF-kB set low
Node ATP 0.70
Node ADP 0.70
Node ATPconsume 0.8
Node Energy_tot 0.0
Node MRSP 0.80
Node ROS 0.1
Node OxProt 0.0
Node ProtBiosynth 0.8
#
```

```
# PROCESSES
#
# 01 Free radical levels
#
Reaction OXROT_ROS 0.1
  pro OxProt 5 5 5 5 5 0
  act ROS
# Protein Turnover
Reaction OxProt_Bio 0.1
  pro OxProt 5 5 5 5 5 0
  inh ProtBiosynth
#
#Reaction ROS scavenged/absorbed
#
Reaction ROS_decay 0.4
 sub ROS 1 2 3 4 5 5
#
#
# 02 Mitochondrial activity
#
# Damage by ROS inhibits mitochondrial respiration
#
# Mitochondrial respiration (MRSP) activated by ADP
Reaction MRSP_activation 0.8
  pro MRSP 5 5 5 5 5 5
  act ADP
# Any damage reduces MRSP
#
Reaction MRSP_inhibition 0.15
  sub MRSP 5 5 5 5 5 5
  act OxProt
Reaction MRSP_ATP 1.0
  pro ATP 5 5 5 5 5 0
  act MRSP
Reaction MRSP_ADP 1.0
  sub ADP 5 5 5 5 5 5
  act MRSP
# ROS generation related to mitochondrial activity and damage
Reaction ROS_produced 0.25
  pro ROS 5 5 5 5 5 0
  act MRSP
Reaction ROS_produced 0.50
  pro ROS 5 5 5 5 5 0
  act OxProt
#
Reaction MRSP_deactivation 0.8
  sub MRSP 5 5 5 5 5 5
  act ATP
#
# Autophagy
#
```

```
Reaction Autophagy_OXPROT 0.15
  sub OxProt 1 2 3 4 5 5
  act ATP
#
# 03 ER Reactions
#
# Biosynth inhibited by oxidized proteins, damage to ER
#
# Production of ADP
Reaction Biosynth_act 1.0
  pro ATPconsume 5 5 5 5 5 0
  act ATP
# Effective Protein Synthesis
Reaction ProteinSynth 0.20
  sub ProtBiosynth 5 5 5 5 5 5
  inh ATPconsume 5 4 3 2 0 0
#
# Inhibition of biosynthesis by damaged ribosomes
#
Reaction Biosynth_OXPROT_inh 0.01
  sub ATPconsume 5 5 5 5 5 5
  act OxProt
Reaction ATP_used 1.0
  sub ATP 5 5 5 5 5 5
  act ATPconsume
Reaction Biosynth_ADP 1.0
  pro ADP 5 5 5 5 5 0
  act ATPconsume
#
# total acc. energy consumed
#
Reaction Energy_totals 0.015
  pro Energy_tot 5 5 5 5 5 5
  act ATPconsume
#
Reaction Biosynth_consumed 1.0
  sub ATPconsume 5 5 5 5 5 5
  act ADP
#
# end of input file
```

Adaptive Response (AR) Model

```
#
#  Adaptive Response Model
#  NF-kB & mTor
#  Jan 5, 2010
#
nFuzzyStates 6

starttime 0.0
endtime 40.0
ntimesteps 10000

PopulationSize 1
nGenerations 1
mutationRate 0.05

Exp

# Define variables to be plotted
#

Plot ROS
# Level of free radicals
Plot OxProt
# Level of oxidized proteins
Plot MRSP
# Mitochondrial respiration
Plot ATP
# ATP levels
Plot Energy_tot
# Total Energy turnover
Plot ADP
# ADP levels
Plot ATPconsume
# Protein Synthesis
Plot ProtBiosynth
# Biosynthesis activity
Plot NFKB
# NFkB levels
Plot MTOR
#Plot of mTor activity
#
# Define nodes, start with initial condition
# ATP and Biosynthesis high, ROS and NF-kB set low
#
Node ATP 0.70
Node ADP 0.70
```

```
Node ATPconsume 0.80
Node ProtBiosynth 0.8
Node Energy_tot 0.0
Node MRSP 0.80
Node NFKB 0.07
Node MTOR 0.4
Node ROS 0.1
Node OxProt 0.0
#
# PROCESSES
#
#
# 01 Free radical levels
#
# ROS causes increase in oxidized proteins
#
Reaction OXPROT_ROS 0.1
  pro OxProt 5 5 5 5 5 0
  act ROS
# Oxidation dependent on protein turnover
Reaction OXPROT_Bio 0.1
  pro OxProt 5 5 5 5 5 0
  inh ProtBiosynth
#
Reaction ROS_decay 0.4
  sub ROS 1 2 3 4 5 5
# NFKB activates scavengers was 0.2
Reaction ROS_scavenge 0.2
  sub ROS 1 2 3 4 5 5
  act NFKB
#
# 02 Mitochondrial activity and ATP generation
#
#
# Buildup of mitochondrial respiration inhibited by ROS
#
# Addtl. ATP generated by glycolysis mediated by mTOR and NF-kB
#
Reaction Glycolysis_ATP 0.75
  pro ATP 5 5 5 5 5 0
  act MTOR 5 3 0 0 0 0
# ADP consumed by Glycolysis
Reaction ADP_Glycolysis 0.75
  sub ADP 0 5 5 5 5 5
  act MTOR 5 3 0 0 0 0
#
Reaction Glycolysis_NFKB_ATP 0.1
  pro ATP 5 5 5 5 5 0
  act NFKB 0 0 1 2 3 4
# ADP consumed by Glycolysis
Reaction ADP_NFKB_Glycolysis 0.1
```

```
  sub ADP 0 5 5 5 5 5
  act NFKB 0 0 1 2 3 4
# Remaining ADP builds potential for mitochondrial respiration
#
Reaction MRSP_activation 0.8
  pro MRSP 5 5 5 5 5 5
  act ADP
#
#
# Downregulation of mitochondrial respiration by stress response
#
Reaction MRSP_change 0.15
  sub MRSP 5 5 5 5 5 5
  act MTOR 2 1 0 0 0 0
Reaction MRSP_change 0.15
  pro MRSP 5 5 5 5 5 5
  act MTOR 0 0 0 1 2 3
#
Reaction MRSP_inhibition 0.15
  sub MRSP 5 5 5 5 5 5
  act OxProt
# NFKB inhibits MRSP
Reaction NFKB_MRSP 0.05
  sub MRSP 5 5 5 5 5 5
  act NFKB
#
Reaction MRSP_ATP 1.0
  pro ATP 5 5 5 5 5 0
  act MRSP
Reaction MRSP_ADP 1.0
  sub ADP 5 5 5 5 5 5
  act MRSP
#
Reaction ROS_produced 0.25
  pro ROS 5 5 5 5 5 0
  act MRSP
Reaction ROS_produced 0.50
  pro ROS 5 5 5 5 5 0
  act OxProt
#
# Autocrine loop NADPH Oxidase System
#
Reaction NADPHOXD_produced 0.1
  pro ROS 5 5 5 5 5 0
  act NFKB
Reaction MRSP_deactivation 0.8
  sub MRSP 5 5 5 5 5 5
 act ATP
#
# NF-kB degradation
#
```

```
Reaction NFKB_decay 0.4
  sub NFKB 0 1 2 3 4 5
#
# 03 Activation of Stress Response
# through ROS levels and oxidized proteins (1:1)
#
Reaction NFKB_Stress_ROS 0.25
   pro NFKB 5 5 5 5 5 0
   act ROS
Reaction NFKB_Stress_OXPROT 0.25
   pro NFKB 5 5 5 5 5 0
   act OxProt
#
# Activation of mTOR
# by low ATP levels
#
# >>>> For 20% less mTOR sensitivity constant is
#          decreased from 0.075 to 0.06
#
Reaction MTOR_ATP 0.075
  sub MTOR 0 1 5 5 5 5
  inh ATP
# Activation of mTOR by ROS
Reaction MTOR_ROS 0.08
  pro MTOR 5 5 5 1 1 0
  act ROS
#
# Autophagy
#
Reaction Autophagy_OXPROT 0.15
sub OxProt 1 2 3 4 5 5
act ATP
# Reaction Autophagy_MTOR 0.05
sub OxProt 1 2 3 4 5 5
act MTOR 5 2 1 0 0 0
#
# 03 ER Reactions
#
# ATP consumption inhibited by oxidized proteins
# damage to ER
#
# Production of ADP
Reaction Biosynth_act 1.0
  pro ATPconsume 5 5 5 5 5 0
 act ATP
#
# Relative Protein Synthesis Scaled
# >>>>>> scaled to be 30% at 60% ATPconsume
#               for lower sensitivity of mTor rate is set to 0.06
#
Reaction ProteinSynth 0.07
```

```
  sub ProtBiosynth 5 5 5 5 5 5
  inh ATPconsume
#
# Oxidative damage to ribosomes
#
Reaction Biosynth_OXPROT_inh 0.01
  sub ATPconsume 5 5 5 5 5 5
  act OxProt
#
# Modulation of ATP consumption regulated by mTor
#
#
Reaction Biosynth_mTOR_inh 0.05
  sub ATPconsume 5 5 5 5 5 5
  act MTOR 5 3 0 0 0 0
#
Reaction Biosynth_mTOR_inh 0.05
  pro ATPconsume 5 5 5 5 5 5
  act MTOR 0 0 0 3 5 0
#
# Biosynthesis required for upregulated inflammatory proteins
#
Reaction Biosynth_NFKB_inflamm 0.05
  pro ATPconsume 5 5 5 5 5 5
  act NFKB
# Final ATP/ADP levels
Reaction ATP_used 1.0
  sub ATP 5 5 5 5 5 5
  act ATPconsume
Reaction Biosynth_ADP 1.0
  pro ADP 5 5 5 5 5 0
  act ATPconsume
#
# total accumulated energy turnover
#
Reaction Energy_totals 0.015
  pro Energy_tot 5 5 5 5 5 5
  act ATPconsume
#
Reaction Biosynth_consumed 1.0
  sub ATPconsume 5 5 5 5 5 5
  act ADP
#
# end of input file
```
