# Supplementary material for: Rule-Based Cell Systems Model of Aging using Feedback Loop Motifs Mediated by Stress Responses
Source: PLoS Comput Biol. 2010 Jun 17;6(6):e1000820. doi: 10.1371/journal.pcbi.1000820 (PMC2887462; doi:10.1371/journal.pcbi.1000820)
Supplement: Table S1 — List of processes and rate constants. (0.08 MB PDF) [file pcbi.1000820.s002.pdf]

Supplemental Table S1: Processes and Rates

Table 1. Processes and reaction rates

Reaction name		Rate CV model	Rate AR model	Description
OXPROT_ROS		0.1	0.1	Protein oxidation by ROS
OXPROT_Bio		0.1	0.1	Oxidation rate is increased by low biosynthesis
ROS_decay		0.4	0.4	ROS decays
ROS_scavenge		0.2	0.2	NFKB activates ROS scavengers
Glycolysis_ATP		NA	.75	Production of ATP by glycolysis depending on mTOR
ADP_Glycolysis		NA	.75	Reduction of ADP if glycolysis active
Glycolysis_NFKB_ATP		NA	.75	Production of ATP by glycolysis depending on NF-KB
ADP_NFKB_Glycolysis		NA	.75	Reduction of ADP if NF-kB glycolysis active
MRSP_activation		0.8	0.8	Mitochondrial respiration is activated by ADP levels
MRSP_change		NA	0.15	Mito-Respiration is inhibited by low mTOR
MRSP_change		NA	0.15	Mito-Respiration is activated by high mTOR
MRSP_inhibition		0.15	0.15	Mito-Respiration is inhibited by oxidized proteins
NFKB_MRSP		NA	.05	Mito-Respiration is inhibited by NFKB
MRSP_ATP		1.0	1.0	ATP is generated by Mito-Respiration
MRSP_ADP		1.0	1.0	ADP is consumed by mito.-respiration
ROS_produced		0.25	0.25	ROS generated by mito-respiration
ROS_produced		0.50	0.50	ROS generated by oxidative damage to mitochondria
NADPHOXD_produced		NA	0.1	NADPH oxidase system is activated and produces

Reaction name		Rate CV model	Rate AR model	Description
				ROS through autocrine loop depending on NFKB
MRSP_deactivation		0.8	0.8	Mito-Respiration is deactivated with reference to ATP produced
NFKB_decay		NA	0.4	Decay of NFKB
NFKB_Stress_ROS		NA	0.25	Activation of NFKB by ROS
NFKB_Stress_OXPROT		NA	0.25	Activation of NFKB by oxidized proteins
MTOR_ATP		0.06	0.06	MTOR is activated by low ATP levels
MTOR_ROS		0.08	0.08	MTOR is activated by high ROS levels
Autophagy_OXPROT		0.15	0.15	Autophagy of oxidized proteins at constant rate
Autophagy_MTOR		NA	0.05	Autophagy of proteins mediated by mTOR
Biosynth_act		1	1	ATP consumption activated by ATP levels
ProteinSynth		0.06	0.06	Protein biosynthesis scaled in relation to ATP consumption
Biosynth_OXPROT_inh		0.01	0.01	ATP consumption inhibited by oxidized proteins
Biosynth_mTOR_inh		NA	0.05	ATP consumption inhibited by low mTOR levels
Biosynth_mTOR_inh		NA	0.05	ATP consumption activated by high mTOR levels
Biosynth_NFKB_inflamm		NA	0.05	Biosynthesis required for inflammatory related proteins
ATP_used		1	1	ATP levels reduced by ATP consumed
Biosynth_ADP		1	1	ADP produced by level of biosynthesis
Energy_totals		0.015	0.015	total accumulated energy turnover
Biosynth_consumed		1	1	ATP consumption determined by biosynthesis
