# Supplementary material for: Rule-Based Cell Systems Model of Aging using Feedback Loop Motifs Mediated by Stress Responses
Source: PLoS Comput Biol. 2010 Jun 17;6(6):e1000820. doi: 10.1371/journal.pcbi.1000820 (PMC2887462; doi:10.1371/journal.pcbi.1000820)
Supplement: Text S1 — Description of the sensitivity analysis. (0.16 MB PDF) [file pcbi.1000820.s003.pdf]

Supplemental Text S1: Sensitivity Analysis

Introduction

The purpose of the sensitivity study for the Adaptive Response model is to examine the effect of changing one input reaction rate at a time, and see what effect it has on the projected fitness of the organism using three criteria. This study examines a +5% change in rate for all reactions, then selects the most significant results from that set to also run -5% and +/- 20% changes.

Criteria

This study uses three (biological) fitness criteria for measuring the fitness of the organism: ATP consumption, ROS levels, and levels of oxidized proteins.

- Depletion of ATPconsume is used as a marker for fitness, since that variable corresponds to the amount of protein synthesis taking place. As a practical matter, the first time step with ATPconsume < 0.4 is used for fitness.
- ROS levels typically start low, and increase throughout life to varying extents. When ROS exceeds 0.4 in the cell defines a second marker for fitness.
- Finally, accumulation of oxidized proteins (OxProt) when exceeding 0.4 is used as the third measure of fitness.

A set of runs were conducted which increase each reaction rate individually by 5% to see what effect that has on reaching fitness according to each of the above three criteria.

Baseline results

The baseline Bionet model data file (AR model), as listed in the Supplemental Data, was executed using the 11/2/2006 version of Bionet. This case establishes the baseline 'fitness' values as follows.

- The first value of ATPconsume < 0.4 is reached at time t=34.17 in the baseline case
- The first value of ROS > 0.4 is reached at t=22.15
- The first value of OxProt > 0.4 is reached at t=28.72.

Input parameters

The model's reactions and their baseline rates are summarized in Table S1. They are listed in the order in which they appear in the data file.

Table S1. Baseline reactions and reaction rates

Reaction name	Baseline rate	Test case
OXPROT_ROS	0.1	Plus01
OXPROT_Bio	0.1	Plus02
ROS_decay	0.4	Plus03
ROS_scavenge	0.2	Plus04
Glycolysis_ATP	.75	Plus05
ADP_Glycolysis	.75	Plus06
MRSP_activation	0.8	Plus07
MRSP_change	0.15	Plus08
MRSP_change	0.15	Plus09
MRSP_inhibition	0.15	Plus10
NFKB_MRSP	.05	Plus11
MRSP_ATP	1.0	Plus12
MRSP_ADP	1.0	Plus13
ROS_produced	0.25	Plus14
ROS_produced	0.50	Plus15
NADPHOXD_produced	0.1	Plus16
MRSP_deactivation	0.8	Plus17
NFKB_decay	0.4	Plus18
NFKB_Stress_ROS	0.25	Plus19
NFKB_Stress_OXPROT	0.25	Plus20
MTOR_ATP	0.075	Plus21
MTOR_ROS	0.08	Plus22
Autophagy_OXPROT	0.15	Plus23
Autophagy_MTOR	0.05	Plus24
Biosynth_act	1	Plus25
ProteinSynth	0.075	Plus26
Biosynth_OXPROT_inh	0.01	Plus27
Biosynth_mTOR_inh	0.05	Plus28
Biosynth_mTOR_inh	0.05	Plus29
Biosynth_NFKB_inflamm	0.05	Plus30
ATP_used	1	Plus31
Biosynth_ADP	1	Plus32
Energy_totals	0.015	Plus33
Biosynth_consumed	1	Plus34

ANALYSIS

The output files from each run (e.g. Plus01.txt.out) were imported into Microsoft Excel 2007. The first time step meeting each criterion given earlier was recorded as the end of the 'lifespan' of the organism. Time resolution was in intervals of 0.01.

The most significant results are given in Table S2. Thirteen of the 34 reactions (38%) were significant, defined as follows:

Let SOF be the Sensitivity Objective Function (Aldridge, 2006), defined as the ratio of the percent change in lifespan with the percent change in the selected reaction rate. This provides a nondimensional way to compare different percent changes in the input functions. A |SOF| > 2.5 is considered a significant sensitivity.

SOF = (% lifespan change) / (% of input parameter change)

ROS lifespan is the most accurate predictor of sensitivity. All reactions that were significant were sensitive to ROS. Most of the significantly sensitive reactions were associated with mitochondrial respiration (MRSP), MTOR, or biosynthesis.

Table S2. Significant Plus 5% change sensitivity results

|SOF| > 2.5 are highlighted as significant

Reaction name	Base rate	Case	SOF-ATPcon	SOF-ROS	SOF-OxProt
ROS_decay	0.4	Plus03	1.2	10.4	7.5
MRSP_activation	0.8	Plus07	10.9	-11.2	-1.9
MRSP_ATP	1	Plus12	4.4	-9.5	-2.9
MRSP_ADP	1	Plus13	18.3	3.5	7.2
ROS_produced	0.25	Plus14	-0.7	-6.9	-2.5
MRSP_deactivation	0.8	Plus17	-5.4	9.2	7.5
MTOR_ATP	0.075	Plus21	0.5	4.7	3.8
MTOR_ROS	0.08	Plus22	-0.4	-4.5	-1.4
Autophagy_OXPROT	0.15	Plus23	0.8	4.7	2.6
Biosynth_act	1	Plus25	9.1	-9.5	-0.9
ATP_used	1	Plus31	9.3	-2.5	0.8
Biosynth_ADP	1	Plus32	4.8	-11.2	-3.7
Biosynth_consumed	1	Plus34	-5.6	8.5	6.1

Table S3 presents the SOF values for reaction rates percent changes of +5%, and values for -5%, +20% and -20% for the most significant thirteen reactions are provided in Table S4, Table S5 and Table S6 respectively.

Table S3. Summary of SOF for a Plus 5% change for every reaction rate

%ATP, %ROS, and %OxProt are the percent changes in lifespan relative to the baseline case.
The SOF columns are highlighted for |SOF| > 2.5

Reaction name	%ATP	%ROS	%OxProt	Case	SOF-ATPcon	SOF-ROS	SOF-OxProt
OXPROT_ROS	-2.4	-5.5	-3.2	Plus01	-0.5	-1.1	-0.6
OXPROT_Bio	-4.4	-11.9	-6.6	Plus02	-0.9	-2.4	-1.3
ROS_decay	5.9	51.9	37.5	Plus03	1.2	10.4	7.5
ROS_scavenge	1.1	10.9	5.2	Plus04	0.2	2.2	1.0
Glycolysis_ATP	-2.3	-11.9	-5.0	Plus05	-0.5	-2.4	-1.0
ADP_Glycolysis	2.8	3.4	12.4	Plus06	0.6	0.7	2.5
MRSP_activation	54.5	-56.0	-9.7	Plus07	10.9	-11.2	-1.9
MRSP_change	-0.6	1.1	0.5	Plus08	-0.1	0.2	0.1
MRSP_change	-0.6	0.1	0.0	Plus09	-0.1	0.0	0.0
MRSP_inhibition	-1.2	3.6	1.7	Plus10	-0.2	0.7	0.3
NFKB_MRSP	-0.7	1.6	0.7	Plus11	-0.1	0.3	0.1
MRSP_ATP	21.8	-47.4	-14.4	Plus12	4.4	-9.5	-2.9
MRSP_ADP	91.4	17.5	35.8	Plus13	18.3	3.5	7.2
ROS_produced	-3.6	-34.6	-12.3	Plus14	-0.7	-6.9	-2.5
ROS_produced	-1.6	-10.9	-3.2	Plus15	-0.3	-2.2	-0.6
NADPHOXD_produced	-1.0	-2.8	-0.9	Plus16	-0.2	-0.6	-0.2
MRSP_deactivation	-26.9	45.8	37.7	Plus17	-5.4	9.2	7.5
NFKB_decay	-0.3	-2.1	-0.7	Plus18	-0.1	-0.4	-0.1
NFKB_Stress_ROS	0.1	4.4	2.2	Plus19	0.0	0.9	0.4
NFKB_Stress_OXPROT	0.0	1.7	0.8	Plus20	0.0	0.3	0.2
MTOR_ATP	2.3	23.6	18.9	Plus21	0.5	4.7	3.8
MTOR_ROS	-1.8	-22.3	-6.8	Plus22	-0.4	-4.5	-1.4
Autophagy_OXPROT	3.9	23.6	12.9	Plus23	0.8	4.7	2.6
Autophagy_MTOR	0.6	5.5	3.0	Plus24	0.1	1.1	0.6
Biosynth_act	45.3	-47.7	-4.7	Plus25	9.1	-9.5	-0.9
ProteinSynth	-2.2	-5.0	-3.9	Plus26	-0.4	-1.0	-0.8
Biosynth_OXPROT_inh	-0.3	1.3	0.7	Plus27	-0.1	0.3	0.1
Biosynth_mTOR_inh	-0.6	2.0	1.0	Plus28	-0.1	0.4	0.2
Biosynth_mTOR_inh	-0.3	0.7	0.4	Plus29	-0.1	0.1	0.1
Biosynth_NFKB_inflamm	0.3	0.0	0.2	Plus30	0.1	0.0	0.0
ATP_used	46.7	-12.4	3.8	Plus31	9.3	-2.5	0.8
Biosynth_ADP	23.8	-56.0	-18.6	Plus32	4.8	-11.2	-3.7
Energy_totals	0.0	1.6	0.9	Plus33	0.0	0.3	0.2
Biosynth_consumed	-27.8	42.4	30.7	Plus34	-5.6	8.5	6.1

Table S4. Summary of SOF for Minus 5% change in selected reaction rates

Reaction name	%ATP	%ROS	%OxProt	Case	SOF-ATPcon	SOF-ROS	SOF-OxProt
ROS_decay	-4.5	-39.6	-13.3	min03	-0.9	-7.9	-2.7
MRSP_activation	-28.1	45.5	37.5	min07	-5.6	9.1	7.5
MRSP_ATP	88.2	12.7	31.1	min12	17.6	2.5	6.2
MRSP_ADP	23.6	-55.9	-18.7	min13	4.7	-11.2	-3.7
ROS_produced	5.8	37.7	31.9	min14	1.2	7.5	6.4
MRSP_deactivation	52.4	-56.1	-9.8	min17	10.5	-11.2	-2.0
MTOR_ATP	-2.0	-25.4	-7.8	min21	-0.4	-5.1	-1.6
MTOR_ROS	1.4	20.0	14.5	min22	0.3	4.0	2.9
Autophagy_OXPROT	-5.1	-13.5	-7.5	min23	-1.0	-2.7	-1.5
Biosynth_act	-27.5	42.2	31.2	min25	-5.5	8.4	6.2
ATP_used	22.4	-47.6	-14.3	min31	4.5	-9.5	-2.9
Biosynth_ADP	56.1	-8.2	9.3	min32	11.2	-1.6	1.9
Biosynth_consumed	48.7	-48.6	-4.8	min34	9.7	-9.7	-1.0

Table S5. Summary of SOF for Plus 20% change in selected reaction rates

Cases marked with * did not 'die' within 100 time units.

Reaction name	%ATP	%ROS	%OxProt	Case	SOF-ATPcon	SOF-ROS	SOF-OxProt
ROS_decay	*	*	126.5	20plus03	*	*	6.3
MRSP_activation	*	-65.3	-13.6	20plus07	*	-3.3	-0.7
MRSP_ATP	39.6	-43.0	-7.8	20plus12	2.0	-2.2	-0.4
MRSP_ADP	55.9	-8.8	7.2	20plus13	2.8	-0.4	0.4
ROS_produced	1.8	-75.5	-28.3	20plus14	0.1	-3.8	-1.4
MRSP_deactivation	-70.4	37.1	25.6	20plus17	-3.5	1.9	1.3
MTOR_ATP	157.4	70.7	64.7	20plus21	7.9	3.5	3.2
MTOR_ROS	13.4	-48.3	-15.7	20plus22	0.7	-2.4	-0.8
Autophagy_OXPROT	18.2	*	*	20plus23	0.9	*	*
Biosynth_act	256.1	-55.5	-4.8	20plus25	12.8	-2.8	-0.2
ATP_used	-32.5	-46.6	-43.1	20plus31	-1.6	-2.3	-2.2
Biosynth_ADP	28.0	-65.6	-24.9	20plus32	1.4	-3.3	-1.2
Biosynth_consumed	-74.5	40.0	18.6	20plus34	-3.7	2.0	0.9

Table S6. Summary of SOF for Minus 20% change in selected reaction rates

Cases marked with * did not 'die' within 100 time units.

Reaction name	%ATP	%ROS	%OxProt	Case	SOF-ATPcon	SOF-ROS	SOF-OxProt
ROS_decay	-0.1	-78.5	-32.8	20min03	0.0	-3.9	-1.6
MRSP_activation	-71.4	37.9	24.8	20min07	-3.6	1.9	1.2
MRSP_ATP	-5.3	-36.4	-31.9	20min12	-0.3	-1.8	-1.6
MRSP_ADP	27.5	-66.8	-26.2	20min13	1.4	-3.3	-1.3
ROS_produced	155.1	74.6	74.0	20min14	7.8	3.7	3.7
MRSP_deactivation	*	-66.2	-13.3	20min17	*	-3.3	-0.7
MTOR_ATP	17.0	-52.4	-16.6	20min21	0.9	-2.6	-0.8
MTOR_ROS	151.8	68.3	58.1	20min22	7.6	3.4	2.9
Autophagy_OXPROT	-16.8	-43.7	-25.6	20min23	-0.8	-2.2	-1.3
Biosynth_act	-75.0	34.3	16.2	20min25	-3.8	1.7	0.8
ATP_used	43.9	-41.2	-5.8	20min31	2.2	-2.1	-0.3
Biosynth_ADP	38.9	-19.3	-6.1	20min32	1.9	-1.0	-0.3
Biosynth_consumed	*	-56.6	-4.5	20min34	*	-2.8	-0.2

Sensitivity analysis with SOF

If the lifespans (ATP consumption, ROS, and OxProt) behaved linearly with respect to a change in one reaction's rate, then the SOF should be constant for various percent changes in that reaction rate. For example, if SOF=2, then a +5% change in rate would produce a 10% increase in lifespan, and a +20% change in rate will produce a 40% increase in lifespan.

Based on the thirteen significant reactions from the +5% sensitivity study, the SOF is summarized in Tables S7-S9 for ATP consumption, ROS, and OxProt respectively. All reactions show nonlinear behavior from +20 to -20 percent changes.

Table S7. ATP consumption SOF as a function of percent change

Percent change in reaction rate

Reaction name	20	5	-5	-20
ROS_decay		1.2	-0.9	0.0
MRSP_activation		10.9	-5.6	-3.6
MRSP_ATP	2.0	4.4	17.6	-0.3
MRSP_ADP	2.8	18.3	4.7	1.4
ROS_produced	0.1	-0.7	1.2	7.8
MRSP_deactivation	-3.5	-5.4	10.5	
MTOR_ATP	7.9	0.5	-0.4	0.9
MTOR_ROS	0.7	-0.4	0.3	7.6
Autophagy_OXPROT	0.9	0.8	-1.0	-0.8
Biosynth_act	12.8	9.1	-5.5	-3.8
ATP_used	-1.6	9.3	4.5	2.2
Biosynth_ADP	1.4	4.8	11.2	1.9
Biosynth_consumed	-3.7	-5.6	9.7	

Table S8. ROS SOF as a function of percent change

Percent change in reaction rate

Reaction name	20	5	-5	-20
ROS_decay		10.4	-7.9	-3.9
MRSP_activation	-3.3	-11.2	9.1	1.9
MRSP_ATP	-2.2	-9.5	2.5	-1.8
MRSP_ADP	-0.4	3.5	-11.2	-3.3
ROS_produced	-3.8	-6.9	7.5	3.7
MRSP_deactivation	1.9	9.2	-11.2	-3.3
MTOR_ATP	3.5	4.7	-5.1	-2.6
MTOR_ROS	-2.4	-4.5	4.0	3.4
Autophagy_OXPROT		4.7	-2.7	-2.2
Biosynth_act	-2.8	-9.5	8.4	1.7
ATP_used	-2.3	-2.5	-9.5	-2.1
Biosynth_ADP	-3.3	-11.2	-1.6	-1.0
Biosynth_consumed	2.0	8.5	-9.7	-2.8

Table S9. Oxidized Proteins (OxProt) SOF as a function of percent change

	Percent change in reaction rate			
**Reaction name**	**20**	**5**	**-5**	**-20**
ROS_decay	6.3	7.5	-2.7	-1.6
MRSP_activation	-0.7	-1.9	7.5	1.2
MRSP_ATP	-0.4	-2.9	6.2	-1.6
MRSP_ADP	0.4	7.2	-3.7	-1.3
ROS_produced	-1.4	-2.5	6.4	3.7
MRSP_deactivation	1.3	7.5	-2.0	-0.7
MTOR_ATP	3.2	3.8	-1.6	-0.8
MTOR_ROS	-0.8	-1.4	2.9	2.9
Autophagy_OXPROT		2.6	-1.5	-1.3
Biosynth_act	-0.2	-0.9	6.2	0.8
ATP_used	-2.2	0.8	-2.9	-0.3
Biosynth_ADP	-1.2	-3.7	1.9	-0.3
Biosynth_consumed	0.9	6.1	-1.0	-0.2

Influence of reaction rate on percent change in lifespan

A linear regression analysis was performed to see if there was a statistically significant relationship between the magnitude of the reaction rates, and their percent changes in lifespan (using ROS > 0.4 as a marker). There was not, as evidenced by Figure S1.

The highest R value for regression was 0.110 for the 34 reactions, slightly under the critical R^2 value of 0.118 required for significance. The interesting trend was that slow reactions tended to have smaller magnitude percent changes, while reaction rates near 1 exhibited a large range of percent changes, producing a fan-shaped distribution of data opening to the right. This trend was observed for all three fitness markers (the other two are not shown).

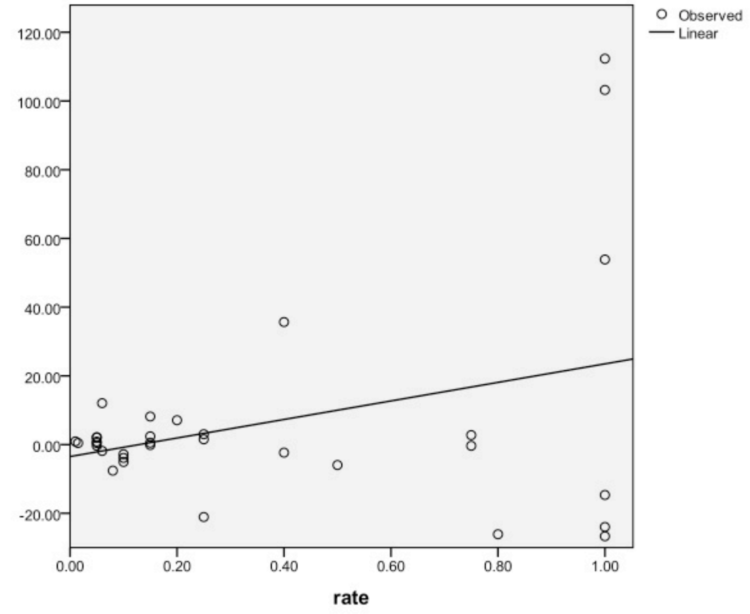

Figure S1. Linear regression of percent change in lifespan versus baseline reaction rates (R^2 = 0.11).
